# Lived Experiences of Iranian Nurses Caring for Brain Death Organ Donor Patients: Caring as “Halo of Ambiguity and Doubt”

**DOI:** 10.5539/gjhs.v8n7p281

**Published:** 2015-12-16

**Authors:** Zahra Keshtkaran, Farkhondeh Sharif, Elham Navab, Sakineh Gholamzadeh

**Affiliations:** 1 Department of Nursing, Student Research Committee, School of Nursing and Midwifery, Shiraz University of Medical Sciences, Shiraz, Iran; 2 Department of Nursing, Community Based Psychiatric Care Research Center, School of Nursing and Midwifery, Shiraz University of Medical Sciences, Shiraz, Iran; 3 Department of Nursing, School of Nursing and Midwifery, Tehran University of Medical Sciences, Tehran, Iran; 4 Department of Nursing, School of Nursing and Midwifery, Shiraz University of Medical Sciences, Shiraz, Iran

**Keywords:** Iranian nurses caring, brain death organ donor, halo of ambiguity and doubt

## Abstract

**Background:**

Brain death is a concept in which its criteria have been expressed as documentations in Harvard Committee of Brain Death. The various perceptions of caregiver nurses for brain death patients may have effect on the chance of converting potential donors into actual organ donors.

**Objective:**

The present study has been conducted in order to perceive the experiences of nurses in care-giving to the brain death of organ donor patients.

**Methods:**

This qualitative study was carried out by means of Heidegger’s hermeneutic phenomenology. Eight nurses who have been working in ICU were interviewed. The semi-structured interviews were recorded by a tape-recorder and the given texts were transcribed and the analyses were done by Van-Mannen methodology and (thematic) analysis.

**Results:**

One of the foremost themes extracted from this study included ‘Halo of ambiguity and doubt’ that comprised of two sub-themes of ‘having unreasonable hope’ and ‘Conservative acceptance of brain death’. The unreasonable hope included lack of trust (uncertainty) in diagnosis and verification of brain death, passing through denial wall, and avoidance from explicit and direct disclosure of brain death in patients’ family. In this investigation, the nurses were involved in a type of ambiguity and doubt in care-giving to the potentially brain death of organ donor patients, which were also evident in their interaction with patients’ family and for this reason, they did not definitely announce the brain death and so far they hoped for treatment of the given patient. Such confusion and hesitance both caused annoyance of nurses and strengthening the denial of patients’ family to be exposed to death.

**Conclusion:**

The results of this study reveal the fundamental perceived care-giving of brain death in organ donor patients and led to developing some strategies to improve care-giving and achievement in donation of the given organ and necessity for presentation of educational and supportive services for nurses might become more evident than ever.

## 1. Introduction

Brain death is a term that has been defined at second half of twentieth century and its criteria were expressed as documents for the first time by Harvard Ad-Hoc Committee of Brain Death in 1968. This term denotes the irreversible conditions and perfect loosing of brain and its stem while there still may be signs of life in patient (Abedi, Mohammadi, & Abdeyazdan, 2012; Akrami Osati, Zahedi, & Raza, 2004; J. Cohen, Ashkenazi, Katvan, & Singer, 2012; Halligan, 2006; Machado, 2007; Wijdicks, 2001; Youngner & Arnold, 2001; Zamperetti Bellomo, Defanti, & Latronico, 2004). Brain death is mainly prevalent in cerebral hypoxia, brain traumas and hemorrhages, and meningitis. These patients are potential good candidates for transplantation of body organs. The results of studies indicate that 1-4% of deaths occur in hospitals and 10% of deaths take place in ICU because of brain death (Azmandian, Shokouhi, Poorhoseini, & Mirzaei, 2013). The different perceptions of nurses of care-giving to brain death patients may affect the chance of converting potential donor of body organs into the actual candidate (Azmandian et al., 2013; Bartucci, 1987; Flodén, Berg, & Forsberg, 2011; Flodén & Forsberg, 2009; Gold, Schulz, & Koch, 2001; Sanner, 2007; White, 2003). Mascia et al. (2009) argue that only 15-20% of brain death patients may be qualified for donation of organs in order to be converted into the actual donors (Mascia, Mastromauro, Viberti, Vincenzi, & Zanello, 2009) and many percent of organs with potentiality for donation are lost due some reasons including inappropriate care and inadequate stabilization during vital period. Hence, the care-giving process is crucial and exact (Azmandian et al., 2013; Jonathan Cohen, Ami, Ashkenazi, & Singer, 2008; Döşemeci, Yılmaz, Cengiz, Dora, & Ramazanoğlu, 2004; Linos Fraser, Freeman, & Foot, 2007; Smith & Vyas, 2011; Vincent, Abraham, Kochanek, Moore, & Fink, 2013). The ICU, brain and nerves units, which receive such patients, are considered as paramount source for donation of organs (Dickerson et al., 2002; Döşemeci et al., 2004; Flodén et al., 2011; Kuo et al., 2006). Transplantation of organs is a relatively new and multidimensional concept and similar to concept of death and several factors may effect on it such as social, cultural, and religious factors (R. Arbour, 2005; Collins, 2005; Manzari et al., 2012; Nasrollahzadeh, Siavosh, & Ghods, 2003). On-time diagnosis of brain death is deemed as the first step in successful management of organ transplantation process and this issue is focused on importance of exact care-giving to brain death patients (Mizraji et al., 2009; Todd, Jerome, & Jarquin-Valdivia, 2007; Vishteh et al., 2010). One of the challenges in today’s medical system of Iran is also fatalities of many needy patients to body organ because of delay in waiting list (Manzari et al., 2012) while more than thousands cases of brain death take place due to several accidents every year and less than 10% of them are donors of body organs. The statistical number of accidents and brain deaths is at high level in Iran so that one death out of every 10 accidents and one case of every 100 deaths is of brain death type (Ahmadian, Haghdoost, & Mohammmmadalizadeh, 2009). Population of Iran is over 77 million while in average eight thousand cases of brain death take place every year. At present, there are about 26 thousands of patients who need the transplantation of body organs, while 7-10 patients die daily due to non- reception of the needed organ. Similarly, according to the latest statistics, the number of donation of organs is 7.8 per million of population in Iran (“News and Information, Ministry of Health and Medical Education,” 2015). Today, brain death has been accepted as the definite and absolute type of death in many developed countries while it is not the same in Asian countries. The studies during two recent decades have shown that there are still noticeable ambiguities regarding brain death among nurses and physicians. In addition, many problems are visible about acceptance of concept of brain death as well (R. B. Arbour, 2012; Bonelli, Prat, & Bonelli, 2009; Joffe, Anton, & Duff, 2012; Pearson, Robertson-Malt, Walsh, & Fitzgerald, 2001; Shewmon, 2009; White, 2003). For instance, John et al (2012), in their studies showed that given the therapeutic care-giver team perceive brain death well, they do not accept it in practice (Jeon et al., 2012). Likewise, the results of investigation done by White (2003) have shown that more than a half of nurses assumed the brain death as type of full death while less than half of them considered the brain death as some conditions such as pending to death and/or incomplete death instead of the stone-dead status (White, 2003) and despite of the existing certain criteria to determine the brain death, one of the greatest concerns, which may so far raise a deep ethical question whether the patient has already died or not. Therefore, the healthcare team may be always afraid, worry and hesitant for isolation of patient from artificial resuscitation system and or to continue it. Such doubts and hesitancies always affect the clinical judgments and process of donation of organs (Bowman & Richard, 2003). Also, Kim et al. (2006) assume ‘the hesitancy of treatment team’ in identifying brain death patient as one of the reasons for shortage of organs transplantation (Kim, Fisher, & Elliott, 2006). Alternately, Flodden et al (2011) in their study have expressed that less than a half of nurses ensure about clinical diagnosis of brain death (Flodén et al., 2011). Today, making effort to increase number of transplantation of organs is considered as the therapeutic preferences throughout the world (Nadoushan et al., 2014). Care-giving to the brain death patient is deemed as an important and crucial effort and it plays vital role in providing the grafted organs. Whereas the subject of transplantation of organs from brain death patients may save the life of other patients therefore it is crucially important and since it is prevalent as a professional activity in different layers of the lived experiences of nurses thus, it is a comprehensible phenomenon. With respect to complexity of dimensions of this phenomenon and in order to perceive deeply the meaning of nurses’ experiences of care-giving to brain death patients as donors of body organs; this study was carried out qualitatively and by taking the hermeneutic phenomenology in order to perceive that how the nurses feel experiences of this phenomenon and how they perceive and interpret it.

## 2. Materials and Methods

Whereas the experiences of caregiver nurses to brain death patients as organ donors are extremely subjective thus the qualitative approach was employed to discover the deep concept of these experiences. Using qualitative research by focusingon Van Manen methodology, we conducted this study since phenomenological studies are suitable for disclosure of the embedded concept in experiences (Creswell, 2012; Grbich, 2012; Navab, Negarandeh, & Peyrovi, 2012; Patton, 2002; Speziale, Streubert, & Carpenter, 2011; Van Manen, 1997, 2001). Based on 6 methodological themes from Van Manen, the following steps were taken:

1) Initially by considering the nature of experience (phenomenon), it was tended to phenomenon of care-giving to brain death patients as organ donors and research questions were formulated. In this study, the research question formed because the researchers have already experienced and concerned with nursing care-giving of brain death patients in ICU ward.
2) At this step, the lived experiences of the participant nurses were continued with inquiry in the given object of experience and focus on discovery of them. In this regard, we studied different texts about the lived experience in different people in the world thereby we could guide our participants and extract the latent concepts in their statements.
3) Our activity was to clarify the internal thematic dimensions of descriptions of participants at this stage. Then, we have separated thematic statements by inter-thematic analysis and after lingual modifications and changes; we eventually extracted the intrinsic and minor themes.
4) At fourth step, researchers presented rich and accurate description and interpretation about the studied experiences through reflection of statements of participants.
5) In the following, we tried to prevent the effect of presuppositions and or involvement from the cycle of pre-classified concepts in our description and interpretation with preservation of string commitment to the research major question which is concerned with the nature of meaning of nurses’ experience in care-giving to brain death patients as organ donors.
6) Finally, through balancing the research platform and considering the parts and whole concept permanently throughout this study; we retracted to the past and presented this text to indicate the meaning of experience (phenomenon) and reviewed this study totally to determine this internal consistency and relevance.

The method of data collection was led by in-depth semi-structured interviews by using the research major question i.e. *‘what is the meaning of caring for brain death organ donor patients?’* Whenever there was any ambiguity in descriptions, it was probed with some questions such as *‘please explain further- what do you mean- and let’s give an example’* in order to achieve clear perception. The interview facilitates rethinking for the researcher along with participants about this experience and perception of its concept and sharing their narrations based on their words (Ajjawi & Higgs, 2007). Initially, the researcher interviewed any interviewee in ICU ward. Interviews were done according to agreement with participants in rest room of ward during hours rather than working shift. Any interview lasted for 45-70 min. Each of 8 participants was interviewed for one session and each of 2 other participants was interviewed for two sessions and totally 12 interviews were executed in association with 10 participants. At the end of each interview, the researcher acknowledged them for sharing their experiences and taking their time. Immediately after each interview, the researcher has transcribed the text of the recorded interviews on tape word-by-word and listened to the interviews for several times and reviewed the transcribed text. The interviews were converted into the enriched texts format of MAXQDA software and entered into this format to facilitate the management. Moreover, in order to collect and interpret data, close and accurate observation technique was employed within the format of methodological and analytical anecdotal note. Accordingly, the researcher in clinical environment directly observed the care-giving behaviors, which were assumed as a part of experiences of nurses with brain death patients as organ donors. At last, the interpretation was presented by the content analysis and via holistic approach and selective approach, meaning units and themes and sub-themes were extracted from interviews.

This study was approved by ethical committee of Shiraz University of Medical Sciences. (Code: EC-93-7005) The studied goals were explained to all of participants and the written consent letter was taken from them. The participants were permitted to exclude from the study if they like. Meanwhile, the researcher offered her phone number and email address to participants for contact and to answer their questions, if any, and any needed information. Likewise, identities of all participants were kept confidential.

Data analysis was done according to the activities at steps 3-6 of Van Mane methodology (2006) as follows:

**Hermeneutic reflection**

Any transcribed text of interviews was read for several times and a general description was written by holistic approach. The thematic analysis was done using phenomenological method. Thematic phrases were separated by the aid of selective approach. The researchers studied the text for several times and they always separated thematic phrases with respect to this main question that what phrases or statements might disclose the nature of the studied phenomenon ‘Care-giving to brain death patient as organ donor’. After highlighting these phrases and statements, they were written as thematic statements. Then, identifying general themes, they were classified and similarities were characterized between texts. Finally, clusters of themes were merged and classified. Interpretation of interviews was accompanied the back and forth movement among the whole and parts and it was done for perception and critical reflection.

**Hermeneutic writing**

The primary findings were discussed hermeneutically between research colleagues. The open visual diversification was used for distinction and approval of major and minor themes.

**Maintaining a strong and oriented relation**

The experiences of participants about care-giving of brain death patients as organ donors were interpreted, described, and perceived.

**Balancing the research context by considering parts and whole**

During research process, researchers retracted to the past frequently and for several times and noticed the context and the whole and analyzed the relation of any part of whole process (Van Manen, 1997).

**Trustworthiness**

To achieve a rigorous study, the researchers implemented all activities based on primary design. Trustworthiness in qualitative researches is based on 4 criteria: Credibility, transferability, confirmability, and dependability (Lincoln & Guba, 1985).

In order to acquire credibility of this study during seminars; the primary findings of research methodology were presented to group of experts and teachers. Following the consideration of the activities relating to credibility and their improvement in qualitative researches, dependability will result. Transferability was achieved by research readers based on general description of rich and deep findings and finally the audit trial of documentations were acquired, which supported the interpretations and dependability. Additionally, themes were described to some participants for checking member.

The participants in this survey (studied population) included nurses from neurosurgery ICU. The inclusion criteria in this study were to have direct experience of care-giving to brain death patient as organ- donor, tendency to participate in the study, and at least 6 months background of working at ICU ward. The research environment in this investigation included the neurosurgery ICU wards at Namaziand Rajayee Hospitals affiliated to Shiraz University of Medical Sciences. It should be noted that Shiraz city is situated at southwestern part of Iran and it is deemed as the transplantation pole at south of Iran and the greatest pole for liver- transplantation in this country. It is impossible to determine sample size in qualitative studies beforehand. Producing of data is continued in this study through the purposeful sampling as long as to achieve rich and deep findings and results. As Van Mannen implies, these data should be rich, deep, abstract, and related (Dinkel, 2005; Kim et al., 2006).

## 3. Results

Our participants included 10 nurses (9 females and 1 male) by the average age of (35.2 yeras) and mean experiences and background (12.4 years) who have participated in this study (Table 1).

**Table 1:** 

Participant	Sex	Age	Level of education	Years of experiences
No 1	Female	29	BS	6
No 2	Female	40	BS	23
No 3	Female	33	MS	9
No 4	Male	25	BS	2
No 5	Female	38	BS	15
No 6	Female	45	BS	25
No 7	Female	43	BS	18
No 8	Female	45	BS	17
No 9	Female	30	BS	6
No 10	Female	25	BS	3

The main theme derived from this study included ‘Halo of ambiguity and doubt’ consisted of two sub-themes of ‘Unreasonable hope’ and ‘Conservative acceptance of brain death’ (Table 2).

**Table 2:** 

Theme	Subthemes	Sub subthemes
Halo of ambiguity & doubt	Unreasonable hope	Expectation for openness (miracle)

	Conservative acceptance of brain death	Lack of confidence to diagnosis and verification of brain death
		Passing through denial wall
		Avoidance from explicit and direct disclosure of brain death to family of patient

The nurses were involved in a type of ambiguity and doubt regarding care-giving of brain death patient as potential organ- donor. This issue was also obvious in their behavior and interaction with the patients’ family. Due to such ambiguity and doubt, they did not definitely announce brain death of patients to their families and still hoped the recovery of the patient. Our participants followed up the patient’s status via phone call even when they were not on duty. Namely, while they assumed pending death for their patients, they did not yet disappointed for recovery of the patients. They did not use term ‘death’ explicitly and directly when they communicated to family of the patient. It seems that such confusion and hesitance about patient’s status and fate cause both bothering the nurses and strengthen the denial of patient’s family in exposure to mourning for their dear patient. These nurses rarely and conservatively accepted brain death and at the same time there was still a track of denial of death of patient(s) in their mind and behavior. In other words, they were so far involved in critical status of this fact that whether patient was dead or alive at last.

**Unreasonable hope**

The nurses as participants in our study always possessed unreasonable and false hope toward patient’s status and they typically expected a miracle. When they communicated with family of patient they used some sentences including ‘*Pin your hope in God; Whatever God wants; pray for patient; God is the high power*’rather than implying indirectly to term of ‘death’.

Nurses also believed in that miracle was not improbable and out of mind and it might take place; therefore, they should hope in God as a beyond power.

*Participant No-8 says*: *I try to explain them what exists as possible as they perceive. I told the family to pray for the patient. A miracle may take place and I also personally wait for a miracle. When I explain it well for them and perceive their conditions, I can see it in their faces that I make every effort for their patient so I feel sense of tranquility. I told them that we give them care exactly but due to status of their patient, the medical team could not make further effort for them. The patient has reached to the stage we are not able to do anything else for them. However, you should have hope in God. Pray for the patient, probably it works. Perhaps a miracle takes place …*

**Expectation for a miracle**

Our nurses talked about the occurrence of a miracle in other patients even for comatose patients and they declared perhaps such an event would take place for brain death as well:

*Participant No-3*: *I still hope the patient probably return to life. If God wants He can do it. Many aggravated comatose patients were returned back to life and became conscious. It is not improbable since we, as Iranians and or Muslims, believe in occurrence of a miracle (openness). It may take place regarding these beliefs. I mean our religious paradigms. I am never disappointed of a patient and I never typically lose my hope to it…*.

Participant No-5 declares: *In fact, there is such a sense that occasionally the people extremely think about a miracle. I feel the same when I have contacts with the patient. Such a thing may strike in our mind that we say we should not be negligent about our task. I say perhaps a miracle may take place either! I tell the visitor it may occur by not in terms of medicine. But you should pin hope in God. I say it may occur but not in terms of medicine; however, God may want to do something. Even in life trend when human thinks to be exposed to a deadlock; there is the same feeling at that point*.

**Conservative acceptance of brain death**

Based on what the participant nurses in our study expressed, care-giving of brain death patient as organ- donor was essentially difficult and deemed as conservative acceptance. Such acceptance strengthened their doubt.

**Lack of confidence (uncertainty) about diagnosis and verification of brain death**

According to viewpoint of nurses, although knowledge and technology has been progressed and they are more complete than the past, it is still partial and relative. Thus, no one can ensure entirely about the clinical diagnoses. Human’s brain is also very complex and crucial and many cerebral events are not yet clearly identifiable. Therefore no one can definitely declare that the brain has been stopped completely and became dead. The presence of some weak and partial motions belonged to spinal reflections might also strengthen and provoke doubts and ambiguities in them. They did not know if they called the patient to whom they gave care as dead or alive person. In other words, they were in doubt about dead or living nature of the patient and in this case, acceptance of brain death and its declaration made this process difficult. For this reason, the nurses also examined the symptoms in the patients in terms of confirmation of brain death to remove their own suspicion and doubt. For example, participant No-2 said:

*I say this patient to whom I give care is patient or dead. I do not know it really! I look at the patient all the time perhaps he/she may breathe sometimes. Often I remove the ventilatorto see if the brain death patient is at the same status or not. Was it confirmed? I may personally checkup the brain death using specific tests… I say perhaps one may think about the level of a tenth of percent that the physicians might make themistake. I personally check the patient again all the times that the physicians said he/she suffers from brain death and I execute the tests on patient as I learned. For instance, they say the patient lack respiration so I remove the ventilator to see it personally. For example, they say his/her pupils show no reaction, the Glasgow Coma Scoreis low… I pinch it to ensure but nothing occurs so that I could accept it but it is really difficult and hard*…

Participant No-8 explains: *Let’s see, it is true the science has advanced and confirmed this fact but not at this level. I do not personally entrust in medicine knowledge. Perhaps we do not know something so far and a miracle may take place! Namely, these parts of brain we thought it does not operate may work. The family of patient is placed behind the door and may pray and expect for miracle. I do it too! I say a miracle may take place at the last moment. Even a part of brain, which we do not know, might be stimulated and start working*.

**Passing through denial wall**

In fact, the nurses in our study were passing through denial wall and they typically denied the death of the patients rather than confrontation with crisis of death and passing over the stages of crisis and both them and the family of patients accepted much conservatively death in addition to considering possible occurrence of a miracle and treated it with doubt and suspicion and this issue submerged them further in halo of ambiguity and doubt.

Participant No-8 says: *I myself think that how it is possible for a young patient to be fallen and no one could do anything for him/her because of brain death. Oh God why it occurred in such a way? Namely everything come to the end! This patient was already working in his/her business and life but now she/he is really struggling with death. It could not believe it because it is very difficult to accept it he/she had thousands of hopes and wishes while currently everything has finished either!*

Participant No-2*: I was very pity and upset and when I work with a brain death patient*,

*I always say that for example if this accident took place for the patient in some other way he/she might be now alive and healthy. Namely, does s/he die? That is so easily! Namely, the brain has completely broke down really! Nothing may be done! Probably God willing and patient became healthy*.

**Avoidance from explicit and direct disclosure of brain death to family of patient**

Participants in this study did not refer to brain death of patient directly and clearly upon communication with family of patient and instead they used phrase of exacerbated status of patient.

For instance, Participant No-2 said: *We do not tell to family of patient that patient suffers from brain death and do not use this word. We say patient is not at good status namely we do not tell it explicitly. We and our colleagues may observe that the physicians do not explicitly tell to attendant of patient that their patient suffers from brain death and they think a little for example about the patient’s status and they may tell them patient is in critical status and we tell it as well…*

In fact, direct reference to brain death might put both nurse and family of patient under difficult conditions in terms of spiritual and emotional status and caused stress and fear in nurses and for this reason they avoided from using this term.

## 4. Discussion

The phrase of ‘Halo of ambiguity and doubt’ included certain conditions and still inappropriate status for the nurses, who gave care to brain death patients as potential organ- donors. During care period, nurses suffered from sense of fear and annoyance because of losing their patient. They were not ensured adequately of diagnosis and verification of brain drain and inflicted by a type of confusion and hesitance. Such ambiguity and doubt might be due to their exposure to a human at the edge of crag of life and pending to fall into mortality and death. Belief in losing patient and imminent possibility of death seemed to be difficult and it was accompanied with a mixture of hope and denial. It was the hope that originated from their religious beliefs and denial in confrontation to death of a human under critical conditions, which acted as a high and firm wall against them to the sky. The participant nurses in our study had a gleam of hope in their hearts based on which probably due to the will and determination of the beyond power the patient may be recovered miraculously. They were afraid of this point to declare clearly and directly brain death to family of patient and at the same time they had some fear that they might not be able to manage emotional reactions of family. For this reason, they avoided from expressing brain death and announcement of absolute disappointment of the patient. Their misunderstanding did not return to their knowledge deficitand rational acceptance but it was related to lack of their emotional acceptance.

The majority of Iranian Muslims in our country is Shiite. In our religion, it has been highly emphasized on preservation of life for humans and moment of death when the soul is isolated from the body is determined by God. Alternately, disappointment from Almighty God is deemed as atheism and a sin. Therefore, we have always learned under unfavorable and despairing conditions to be hopeful as well so occurrence of miracle and extraordinary accident will not be improbable and out of our expectation. The media may be also effective in our cultural context in this regard. In our study, the participant nurses expressed that ‘*as usual in TV movies and serials a miracle may entirely change everything under perfect despairing conditions at the eleventh hour and one should typically expect for occurrence a miracle. Therefore, we possess some resistance in acceptance of brain death and end of patient’s life to accept it with difficulty and conservatively under the influence of cultural and religious beliefs*.’ Of course, ambiguity and doubt may put our nurses under the condition that both causes their annoyance and strengthens the experience for denial in family of patient with brain death since perception, acceptance, and behavior of our nurses may effect on perceptions and acceptance of their families and making decision. The presence of a trace of denial in our nurses and doubt and suspicion about this point that if the patient for whom they give care is dead or alive will certainly strengthen family’s denial versus acceptance of death of their dear patient. When our nurses do not explicitly talk about the death and stopping the activity of brain of patients to their family and they express some sentences such as ‘your patient is at the worst status’ and or ‘we could not do anything for your patient but you should hope to God’; ‘whatever God wants’, and ‘pray for your patient’; doubtlessly, such idea may be created in mind of patient’s family that there may be some hope for remedy. Bowman (2003) argues that no one could assume the death only as a biologic event, but it includes some cultural, legal, and political aspects as well (Bowman & Richard, 2003). Also Cheraghi (2005) expresses that passing through death process and its phases depend on religious beliefs of the individuals (Cheraghi, Payne, & Salsali, 2005). Oliver et al. (2012) claim that despite of efforts made by Islamic scientists to improve rate of donation of body organs, many Muslims may be still declined to accept this concept and it seems that the Asian Muslims are more declined to this fact compared to Arab Muslims (Oliver, Ahmed, & Woywodt, 2012).

Watson (2010), has referred to the existing mysterious aspects of death and their dimensions and stated that the nurses should express their openness toward occurrence of miracle and take it into consideration. Kesselring (2007) implies that uncertainty of death moment might be effective on ambiguity and doubt about brain death. It has been referred in his study that the behavior of personnel might affect on intensification of ambiguity and shock of patient’s family in exposure to death of their patient (Kesselring, Kainz, & Kiss, 2007). Of course, it has been referred to the presence of misunderstanding that is assumed as a risk for perception and acceptance of brain death in family of the patient while this problem is also visible in nursing personnel at our study.

Pearson et al. (2001) maintain that perhaps the physical status of patient that is similar to other patients and the care given by the nurses to them similar to other patients based on their professional, ethical, and emotional obligations may exacerbate their doubts regarding acceptance and verification of brain death in patient (Pearson et al., 2001). Cohen (2008) argues that occurrence of confusion about determination and verification of brain death and difference about death concepts between healthcare professions may be followed by some consequences in process of providing donated organs (Jonathan Cohen et al., 2008). We believe that presence of such a halo of ambiguity and doubt among our participants may be also returned to a type of professional ethical challenges in ICU ward since the nature of this task in this ward may expose the nurses to major challenges including ethical dilemma, which often lead to occurrence of confusion and doubt. The fact that the nurses are giving care to the patient, who is seemingly alive but s/he is really dead and possesses some potentially living organs that can save the life of other patients while giving any care is deemed practically as in vain and useless may put them in an ethical challenge and lead them to a type confusion, ambiguity, and doubt. In fact, it is the last alternative that the patient has died and there is no hopes for his/her return to life and recovery?! This question is always unresolved and creates doubt and suspicion about a patient whose heart still beats and it avoids from stating definitely about death. In fact, all of religious and cultural beliefs as well as ethical and emotional conflicts prevail over knowledge and scientific and practical teachings of our nurses and prevent from acceptance of death in patient absolutely. Rittner et al. (2003) state that it is clear that donation of a living organ always raises this ethical question that if this action is against medical ethos or not (i.e. no harm) (Bernat, 2005; Rittner, Besold, & Wandel, 2003). Flodden (2011) expresses that understanding of brain death requires rational and emotional perception and it necessitates for nurses to deal with this concept and transparency of their attitude and perception of care-giving to the brain death patient as potential organ-donor (Flodén et al., 2011).

In a study done by Araujo (2014), the participants also believed that brain death patient might rescue the life of other patients but as long as his/her heart beats the patient should receive care similar to other patients and the patient is deemed as alive for care-giving team as long as s/he is placed in ICU ward and his/her heart beats. Working in such a platform of technology and permanent advancements has caused the nurses and physicians to be put under pressure among knowledge and culture. The ethical conflicts experienced by nurses during care-giving process are assumed as a factor to accept brain death with difficulty in this study. Unlike our study, the participant nurses in their investigation suffered from lack of obligation, knowledge, and omission and negligence in care-giving but other challenges experienced by the nurses were similar to our study including shortage of equipment and human resources and religious influence (Araújo & Massarollo, 2014).

According to the nurses as participants in our study, whatever knowledge and technology advances it will be more perfect than the past but it is incomplete and partial in respective of the future and with respect to medical science and knowledge no one could entirely ensure of medical diagnoses. Alternately, brain is a very complex and crucial organ and many of cerebral activities are not yet completely clear so no one can absolutely deny any type of brain activity and thereby to affix label of absolute death and permanent stoppage of this organ. Of course, the presence of some reflexive motions relating to spinal cord is also considered as another factor that intensifies the doubt and suspicion of nurse to verify brain death and reduces the trust in diagnosis of brain death so that of course they indicate the requisite for acquisition of further scientific information. In his survey, Bernat (2005) has implied that the evidences signify that some physicians may either not properly do the tests for verification of brain death and or record them well and despite of existing conducted and known tests regarding diagnosis and verification of brain death, the only thing that physicians sufficed with it is apnea test (Bernat, 2005).

Based on what it has been so far mentioned, this article reports one of the important themes of or phenomenological study while the findings of our study show both positive and negative aspects of care-giving to brain death patient as potential donor of living organ. ‘Halo of ambiguity and doubt’ has mainly referred to negative aspects of this phenomenon.

## 5. Limitation

The present study was carried out on the limited numbers of caregivers. The small sample size and nature of research may restrict the potentiality to generalize the results. In any case, in all of the qualitative studies, the objective is not generalization of the results.

## 6. Conclusion

The results of this study may add to the body of existing knowledge in this field and disclose the fundamental perceptions of care-giving to these patients. The perceived experience by caregivers, their emotions, requirements, and problems may lead to developing supportive strategies for the patients and presentation of educational and supportive services for the nurses.

**Relevance to Clinical Practice**

The nurses are assumed as the frontline of care-giving to brain death organ donor patients. Our study emphasizes on the necessity for training and employment of nurses along with suitable perceptions in ICUs and transplantation teams. This is an important issue for us to improve the knowledge and information of nursing personnel regarding the method of exposure and better and more favorable management in care-giving to such patients. Training of strategies, which may contribute our nurses in establishing efficient and proper communication with family of brain death organ donor patients in management the final moments of life more than ever and it can reduce difficulty of this stressful situation. Using the results of this study, we may correct the existing misunderstandings about brain death and thereby to upgrade the donation process more successfully.
